# Supplementary material for: An evaluation of factors that may influence clinicians’ decisions not to enroll eligible patients into randomized trials in critical care
Source: PLoS One. 2021 Jul 27;16(7):e0255361. doi: 10.1371/journal.pone.0255361 (PMC8315530; doi:10.1371/journal.pone.0255361)
Supplement: S1 File — (PDF) [file pone.0255361.s001.pdf]

S1 File: List of participating sites

Title: An evaluation of factors that may influence clinicians' decisions not to enrol eligible patients into randomized trials in critical care.

Authors: Mahesh Ramanan, Laurent Billot, Dorrilyn Rajbhandari, John Myburgh and Balasubramanian Venkatesh

List of participating sites:

Auckland City Hospital DCCM

Auckland City Hospital CV ICU

Austin Hospital ICU

Bendigo Hospital ICU

Blacktown Hospital ICU

Calvary Mater Newcastle ICU

Christchurch Hospital ICU

Fiona Stanley Hospital

Fremantle Hospital ICU

Geelong Hospital ICU

Gold Coast University Hospital ICU

Gosford Hospital ICU

Ipswich Hospital ICU

John Hunter Hospital ICU

Liverpool Hospital ICU

Logan Hospital ICU

Lyell McEwin Hospital ICU

Mackay Base Hospital ICU

Mater Adults Hospital (Brisbane) ICU

Mater Misericordiae Hospital ICU

Middlemore Hospital ICU

Monash Medical Centre ICU

Nambour Hospital ICU

Nepean Hospital ICU

North Shore Hospital (NZ) ICU

Northern Hospital

The Prince Charles Hospital ICU

Prince of Wales Hospital (Sydney) ICU

Princess Alexandra Hospital ICU

The Queen Elizabeth Hospital (Adelaide) ICU

Redcliffe Hospital ICU

Royal Brisbane and Women’s Hospital ICU

Royal Adelaide Hospital ICU

Royal Darwin Hospital ICU

Royal Hobart Hospital ICU

Royal Melbourne Hospital ICU

Royal North Shore Hospital ICU

Royal Perth Hospital ICU

Royal Prince Alfred Hospital ICU

St George Hospital ICU

St John of God Hospital ICU

St Vincent’s Hospital – Sydney ICU

St Vincent’s Hospital – Melbourne ICU

Sunshine Hospital ICU

Tauranga Hospital ICU

Tamworth Hospital ICU

Tweed Heads Hospital ICU

Toowoomba Hospital ICU

Townsville Hospital ICU

Waikato Hospital ICU

Wellington Hospital ICU

Wesley Hospital ICU

Western Hospital (Footscray) ICU

Wollongong Hospital ICU

Ashford and St Peter’s Hospital ICU

Freeman Hospital (Newcastle upon Tyne) ICU

Guy’s and St Thomas’ Hospital ICU

King Abdulaziz Medical City Hospital ICU

King Fahad Medical City Hospital ICU

King Khalid University Hospital ICU

King’s College Hospital ICU

Lewisham Healthcare Trust ICU

Queen Alexandra Hospital (Portsmouth) ICU

Queen Elizabeth Hospital (Birmingham) ICU

Rigshospitalet ICU

Royal Gwent Hospital ICU

Royal Surrey County Hospital ICU

Southampton General Hospital ICU

St George’s Healthcare Trust ICU

University Hospitals Bristol ICU
