# Supplementary material for: An evaluation of factors that may influence clinicians’ decisions not to enroll eligible patients into randomized trials in critical care
Source: PLoS One. 2021 Jul 27;16(7):e0255361. doi: 10.1371/journal.pone.0255361 (PMC8315530; doi:10.1371/journal.pone.0255361)
Supplement: Attachment — Submitted filename: PONE-D-21-04343-1 [suggeted edits].docx [file pone.0255361.s002.docx]

PLOS ONE

**An evaluation of factors that may influence clinicians’ decisions not to enroll eligible patients into randomized trials in critical care.**

--Manuscript Draft--

| **Manuscript Number:** | PONE-D-21-04343 |
| --- | --- |
| **Article Type:** | Research Article |
| **Full Title:** | An evaluation of factors that may influence clinicians’ decisions not to enroll eligible patients into randomized trials in critical care. |
| **Short Title:** | Clinicians declining to enroll eligible patients into randomized trials in critical care. |
| **Corresponding Author:** | Mahesh Ramanan, BSc MBBS MMed FCICM Caboolture Hospital  Caboolture, QLD AUSTRALIA |
| **Keywords:** | randomized trials; recruitment; critical care; sepsis; randomization |
| **Abstract:** | Objectives  To determine the association between intensive care unit (ICU) characteristics and clinicians’ decision to decline eligible patients for randomization into a multicentered pragmatic comparative-effectiveness controlled trial.  Methods  Screening logs from the Adjunctive Glucocorticoid Therapy in Septic Shock Trial (ADRENAL) and site-level data from the College of Intensive Care Medicine and Australia New Zealand Intensive Care Society were examined. The effects of ICU characteristics such as tertiary academic status, research coordinator availability, number of admissions, and ICU affiliations on clinicians declining to randomize eligible patients were calculated using mixed effects logistic regression modelling.  Results  There were 21,818 patients screened for inclusion in the ADRENAL trial at 69 sites across five countries, out of which 5,501 were eligible, 3,800 were randomized and 659 eligible patients were declined for randomization by the treating clinician. The proportion of eligible patients declined by clinicians at individual ICUs ranged from 0 to  0.41. In the multivariable model, none of the ICU characteristics were significantly associated with higher clinician decline rate.  Conclusions  Neither tertiary academic status, nor other site-level variables were significantly associated with increased rates of clinicians declining eligible patients. |
| **Order of Authors:** | Mahesh Ramanan, BSc MBBS MMed FCICM |
|  | Laurent Billot |
|  | Dorrilyn Rajbhandari |
|  | John Mybrugh |
|  | Balasubramanian Venkatesh |
| **Opposed Reviewers:** |  |
| **Additional Information:** |  |
| **Question** | **Response** |
| **Financial Disclosure** | The author(s) received no specific funding for this work. |

*Powered by Editorial Manager® and ProduXion Manager® from Aries Systems Corporation*

| Enter a financial disclosure statement that describes the sources of funding for the work included in this submission. Review the [submission guidelines](http://journals.plos.org/plosone/s/submission-guidelines#loc-financial-disclosure-statement) for detailed requirements. View published research articles from [*PLOS ONE*](http://journals.plos.org/plosone/) for specific examples.  This statement is required for submission and **will appear in the published article** if the submission is accepted. Please make sure it is accurate. |  |
| --- | --- |
| **Unfunded studies**  Enter: *The author(s) received no specific funding for this work.*  **Funded studies**  Enter a statement with the following details:   - Initials of the authors who received each award - Grant numbers awarded to each author - The full name of each funder - URL of each funder website - Did the sponsors or funders play any role in the study design, data collection and analysis, decision to publish, or preparation of the manuscript? - **NO** - Include this sentence at the end of your statement: *The funders had no role in study design, data collection and analysis, decision to publish, or preparation of the manuscript.* - **YES** - Specify the role(s) played. |  |
| * typeset |  |
| **Competing Interests**  Use the instructions below to enter a competing interest statement for this submission. On behalf of all authors, disclose any [competing interests](http://journals.plos.org/plosone/s/competing-interests) that could be perceived to bias this work—acknowledging all financial support and any other relevant financial or non- financial competing interests.  This statement is required for submission and **will appear in the published article** if the submission is accepted. Please make | The authors have declared that no competing interests exist. |

*Powered by Editorial Manager® and ProduXion Manager® from Aries Systems Corporation*

| sure it is accurate and that any funding sources listed in your Funding Information later in the submission form are also declared in your Financial Disclosure statement.  View published research articles from  [*PLOS ONE*](http://journals.plos.org/plosone/) for specific examples. |  |
| --- | --- |
| **NO authors have competing interests**  Enter: *The authors have declared that no competing interests exist.*  **Authors with competing interests**  Enter competing interest details beginning with this statement:  *I have read the journal's policy and the authors of this manuscript have the following competing interests: [insert competing interests here]* |  |
| * typeset |  |
| **Ethics Statement**  Enter an ethics statement for this submission. This statement is required if the study involved:   - Human participants - Human specimens or tissue - Vertebrate animals or cephalopods - Vertebrate embryos or tissues - Field research   Write "N/A" if the submission does not require an ethics statement.  General guidance is provided below. Consult the [submission guidelines](http://journals.plos.org/plosone/s/submission-guidelines#loc-guidelines-for-specific-study-types) for detailed instructions. **Make sure that all information entered here is included in the Methods section of the manuscript.** | Institutional Review Board approval was obtained from The Prince Charles Hospital Human Research Ethics Committee (LNR/2018/QPCH/44975). Requirement for consent was waived as the data were anlaysed in a de-identified format. |

*Powered by Editorial Manager® and ProduXion Manager® from Aries Systems Corporation*

| **Format for specific study types**  **Human Subject Research (involving human participants and/or tissue)**   - Give the name of the institutional review board or ethics committee that approved the study - Include the approval number and/or a statement indicating approval of this research - Indicate the form of consent obtained (written/oral) or the reason that consent was not obtained (e.g. the data were analyzed anonymously)   **Animal Research (involving vertebrate animals, embryos or tissues)**   - Provide the name of the Institutional Animal   Care and Use Committee (IACUC) or other relevant ethics board that reviewed the study protocol, and indicate whether they approved this research or granted a formal waiver of ethical approval   - Include an approval number if one was obtained - If the study involved *non-human primates*, add *additional details* about animal welfare and steps taken to ameliorate suffering - If anesthesia, euthanasia, or any kind of animal sacrifice is part of the study, include briefly which substances and/or methods were applied   **Field Research**  Include the following details if this study involves the collection of plant, animal, or other materials from a natural setting:   - Field permit number - Name of the institution or relevant body that granted permission |  |
| --- | --- |
|  |  |
| **Data Availability**  Authors are required to make all data underlying the findings described fully available, without restriction, and from the time of publication. PLOS allows rare exceptions to address legal and ethical concerns. See the [PLOS Data Policy](http://journals.plos.org/plosone/s/data-availability) and [FAQ](http://journals.plos.org/plosone/s/data-availability#loc-faqs-for-data-policy) for detailed information. | No - some restrictions will apply |

*Powered by Editorial Manager® and ProduXion Manager® from Aries Systems Corporation*

| A Data Availability Statement describing where the data can be found is required at submission. Your answers to this question constitute the Data Availability Statement and **will be published in the article**, if accepted. |  |
| --- | --- |
| **Important:** Stating ‘data available on request from the author’ is not sufficient. If your data are only available upon request, select ‘No’ for the first question and explain your exceptional situation in the text box. |  |
| Do the authors confirm that all data underlying the findings described in their manuscript are fully available without restriction? |  |
| **Describe where the data may be found in full sentences. If you are copying our sample text, replace any instances of XXX with the appropriate details.**   - If the data are **held or will be held in a public repository**, include URLs, accession numbers or DOIs. If this information will only be available after acceptance, indicate this by ticking the box below. For example: *All XXX files are available from the XXX database (accession number(s) XXX, XXX.*). - If the data are all contained **within the manuscript and/or Supporting Information files**, enter the following: *All relevant data are within the manuscript and its Supporting Information files.* - If neither of these applies but you are able to provide **details of access elsewhere**, with or without limitations, please do so. For example:   *Data cannot be shared publicly because of [XXX]. Data are available from the XXX Institutional Data Access / Ethics Committee (contact via XXX) for researchers who meet the criteria for access to confidential data.*  *The data underlying the results presented in the study are available from (include the name of the third party* | Data can be made available on request pending approvals from the relevant Institutional Review Board, the Management Committee of the Australia New Zealand Intensive Care Society Centre for Outcomes and Resource Evaluation (as outline here: [https://www.anzics.com.au/data-access-and-publication-policy/](http://www.anzics.com.au/data-access-and-publication-policy/) ), and The George Institute for Global Health (as outlined here: [https://www.georgeinstitute.org/data-](http://www.georgeinstitute.org/data-) sharing-policy ) |

*Powered by Editorial Manager® and ProduXion Manager® from Aries Systems Corporation*

| *and contact information or URL).*   - This text is appropriate if the data are owned by a third party and authors do not have permission to share the data.   * typeset |  |
| --- | --- |
| Additional data availability information: |  |

*Powered by Editorial Manager® and ProduXion Manager® from Aries Systems Corporation*

Cover Letter

8 February 2021

From:

Dr. Mahesh Ramanan

Intensive Care Unit, Caboolture Hospital Caboolture, Queensland, 4510, Australia Email: [Mahesh.ramanan@health.qld.gov.au](mailto:Mahesh.ramanan@health.qld.gov.au)

To:

The Editorial Board PLOS ONE

Dear Editor,

We would like to submit our manuscript entitled “An evaluation of site-level variables that influence clinicians’ decisions not to enrol eligible patients into randomized trials in critical care” for publication as a Research Article in PLOS ONE.

Slow recruitment remains a significant challenge to the conduct of successful randomized trials in critical care medicine. One infrequently studied contributor to trial recruitment challenges is the phenomenon of clinicians declining to randomize eligible patients into trials.

In this manuscript, we have described the clinician decline rate and studied the influence of site-level factors using data from the largest septic shock trial (to date), the “*Adjunctive glucocorticoid therapy in patients with septic shock”* trial (ADRENAL). We have linked site-level data from a high-quality resourcing registry, the Critical Care Resources registry maintained by the Australia New Zealand Intensive Care Society.

This manuscript, nor any other manuscript related to this work, has not been published or posted elsewhere.

We suggest Ludhmila Hajjar or Andrea Cortegiani as appropriate Academic Editors to handle this manuscript.

We look forward to hearing the outcome of your review and would be happy to provide any further information.

Yours sincerely,

Mahesh Ramanan on behalf of all authors

[Click here to access/download;Manuscript;Manuscript_Clinician decline](https://www.editorialmanager.com/pone/download.aspx?id=28362997&guid=1a2d2012-3001-46ae-9e5e-8c448cdfb1eb&scheme=1)

An evaluation of factors that may influence clinicians’ decisions not to enroll eligible patients into randomized trials in critical care.

Mahesh Ramanan^1,2,3,4^, Laurent Billot^2^, Dorrilyn Rajbhandari^2^, John Mybrugh^2^ and Balasubramanian Venkatesh^2,4,5,6^.

1. Intensive Care Unit, Caboolture Hospital, Brisbane, Queensland, Australia
2. Critical Care Division, The George Institute for Global Health, University of New South Wales, Sydney, Australia
3. Adult Intensive Care Services, The Prince Charles Hospital, Brisbane, Queensland, Australia
4. School of Medicine, University of Queensland, St Lucia, Queensland, Australia
5. Intensive Care Unit, Wesley Hospital, Auchenflower, Queensland, Australia
6. Intensive Care Unit, Princess Alexandra Hospital, Woolloongabba, Queensland, Australia

Corresponding Author: Mahesh Ramanan Email: [Mahesh.ramanan@health.qld.gov.au](mailto:Mahesh.ramanan@health.qld.gov.au)

Contributorship: MR and BV conceived and designed the study, performed the analyses, and wrote the manuscript. DR maintained and curated the data. LB, DR and JM assisted in designing aspects of the study and critical review of the manuscript. LB directed the statistical analyses.

# Abstract

Objectives: To determine the association between intensive care unit (ICU) characteristics and clinicians’ decision to decline eligible patients for randomization into a multicentered pragmatic comparative-effectiveness controlled trial.

Methods: Screening logs from the Adjunctive Glucocorticoid Therapy in Septic Shock Trial (ADRENAL) and site-level data from the College of Intensive Care Medicine and Australia New Zealand Intensive Care Society were examined. The effects of ICU characteristics such as tertiary academic status, research coordinator availability, number of admissions, and ICU affiliations on clinicians declining to randomize eligible patients were calculated using mixed effects logistic regression modelling.

Results: There were 21,818 patients screened for inclusion in the ADRENAL trial at 69 sites across five countries, out of which 5,501 were eligible, 3,800 were randomized and 659 eligible patients were declined for randomization by the treating clinician. The proportion of eligible patients declined by clinicians at individual ICUs ranged from 0 to 0.41. In the multivariable model, none of the ICU characteristics were significantly associated with higher clinician decline rate.

Conclusions: Neither tertiary academic status, nor other site-level variables were significantly associated with increased rate of clinicians declining eligible patients.

# Background

Prompt enrollment of eligible patients into randomized controlled trials (RCTs) is recognized as a research priority(1). Enrolment of patients into RCTs in critical care poses challenges including difficulties in obtaining consent because of their altered conscious state due to either underlying conditions or acute therapeutic interventions(2,3). Within this environment, attending clinicians may decline to enroll potentially eligible patients, which has been reported to occur for 3-15% of eligible patients (3,4).

A detailed evaluation of the influence of site-level factors on the clinician decline rate, that is the proportion of eligible patients that are declined for randomization by the treating clinician, in critical care RCTs has not been performed. Some reasons provided by clinicians relate to the nature of the intervention and lack of equipoise. We hypothesized that factors such as the academic and training status of the intensive care unit (ICU) and availability of dedicated research staff may be associated with reduced clinician decline rate.

We performed this study to determine associations between ICU characteristics and clinician decline rate from a large-scale, investigator-initiated, pragmatic, international RCT.

# Methods

Institutional Review Board approval was obtained from The Prince Charles Hospital Human Research Ethics Committee (LNR/2018/QPCH/44975).

Patient randomization data was extracted from the *Adjunctive glucocorticoid therapy in patients with septic shock*(5) (ADRENAL) screening log and linked to ICU characteristics obtained from the Critical Care Resources registry maintained by Australia New Zealand Intensive Care Society (ANZICS) Centre for Outcome and Resource Evaluation. For non-

Australia and New Zealand sites, these data were extracted from the site selection and feasibility log. The screening log contained date and site at which each patient was screened, whether they were eligible, randomized, and reason for not being randomized. We defined tertiary ICU status as any ICU meeting criteria for a Level III ICU as per College of Intensive Care Medicine of Australia and New Zealand (6).

The primary outcome was a binary variable indicating whether an eligible patient was declined for randomization by the treating clinician and was analyzed using a multivariable mixed effects logistic regression model. The clinician decline rate was calculated as the proportion of eligible patients who were declined for enrolment by the treating clinician and was presented as median and interquartile range (IQR). Univariate analyses comparing characteristics of tertiary and non-tertiary sites were performed using Mann-Whitney U test and Fishers exact test for continuous and categorical variables. ICU-level variables (volume, i.e., number of admissions per annum; research coordinator full-time equivalent (FTE); after-hours research coordinator availability; dedicated director of research FTE; and ANZICS Clinical Trials Group membership) were entered as fixed effects and site as random effect, with patients nested within sites. Two models were created; one for Australian and New Zealand sites only (additional covariates were available for these sites) and one including all sites. The effect of the covariates on the risk of being declined for randomization was presented as an adjusted odds ratio (OR) with 95% confidence interval (CI) and p-value

<0.05 considered significant.

# Results

The ADRENAL trial screened 21,818 patients from 69 sites, of whom 5,501 were eligible for randomization, and 3 800 were randomized. The characteristics of tertiary and non-tertiary ICUs

participating in the trial are listed in Table 1. Of the 1,701 patients who were eligible but not randomized, 659 were declined for randomization by the treating clinician. Clinician decline rates ranged from 0 to 0.41,(median rate of 0.07, IQR 0.02-0.19 for tertiary sites and

0.09, IQR 0-0.20 for non-tertiary sites).

In the multivariable analysis (Table 2), tertiary status was not associated with a significantly reduced clinician decline rate (OR 0.89, 95% CI 0.35-2.31, p=0.8 for all sites model; OR 0.52, 95% CI 0.17-1.59, p=0.3 for Australia and New Zealand sites model). None of the other available covariates were significantly associated with clinician decline rate in either model.

# Discussion

The decision by a clinician to exclude a potentially eligible patient is a recognized cause of reduced enrolment into a clinical trial. Using a representative international, large-scale pragmatic RCT in critically ill patients, we report that nearly twelve percent of eligible patients were declined for enrolment by treating clinicians. This rate was not influenced by the academic status of the ICU nor other site characteristics. The ADRENAL trial was a pragmatic study evaluating a commonly used intervention and also allowed deferred consent (patients were allowed to be randomized into the trial and consent obtained subsequently). This suggests that the clinician’s decision is made on a case-by-case basis and unlikely to be due to the nature of the intervention or difficulties in obtaining consent.

Whilst the causes of clinicians declining to enroll eligible patients were not collected in the screening log, we can only speculate that clinicians declined to enroll primarily due of lack of	equipoise.

Whilst RCTs have traditionally been conducted in large tertiary centers(7), our results show that the participation of smaller non-tertiary centers not only add to patient enrollment, but have similar, comparable recruitment patterns.

We acknowledge that this study is limited by a lack of power, as suggested by the wide confidence intervals in the multivariable analysis.

Further investigation into the factors influencing clinician decline rate is warranted. We recommend that screening logs collect additional data to include patient-related, intervention-related, consent-related, or clinician decision factors that lead to eligible patients not being randomized into clinical trials. The establishment of a ‘baseline’ clinician decline rate (due to non-modifiable factors) may be useful in benchmarking the performance of RCTs as well as individual trial sites.

Monitoring the clinician decline rate over the course of a trial may inform trial management committees to address this issue on a real-time basis, so that interventions such as increased frequency and intensity of academic detailing with frequent educational site visits can be effectively targeted.

# Conclusion

Clinicians declining to randomize patients into critical care RCTs has a substantive effect on patient enrollment, but this does not appear to be associated with individual site characteristics.

# Acknowledgements

The authors thank the College of Intensive Care Medicine of Australia and New Zealand, the Australia New Zealand Intensive Care Society Centre for Outcomes and Resource Evaluation management committee, and clinicians, data collectors, and researchers at the following contributing sites:

Auckland City Hospital DCCM Auckland City Hospital CV ICU Austin Hospital ICU

Bendigo Hospital ICU Blacktown Hospital ICU Calvary Mater Newcastle ICU Christchurch Hospital ICU Fiona Stanley Hospital Fremantle Hospital ICU Geelong Hospital ICU

Gold Coast University Hospital ICU Gosford Hospital ICU

Ipswich Hospital ICU John Hunter Hospital ICU

Liverpool Hospital ICU Logan Hospital ICU

Lyell McEwin Hospital ICU Mackay Base Hospital ICU

Mater Adults Hospital (Brisbane) ICU Mater Misericordiae Hospital ICU Middlemore Hospital ICU

Monash Medical Centre ICU Nambour Hospital ICU Nepean Hospital ICU

North Shore Hospital (NZ) ICU Northern Hospital

The Prince Charles Hospital ICU Prince of Wales Hospital (Sydney) ICU Princess Alexandra Hospital ICU

The Queen Elizabeth Hospital (Adelaide) ICU Redcliffe Hospital ICU

Royal Brisbane and Women’s Hospital ICU Royal Adelaide Hospital ICU

Royal Darwin Hospital ICU Royal Hobart Hospital ICU

Royal Melbourne Hospital ICU Royal North Shore Hospital ICU Royal Perth Hospital ICU

Royal Prince Alfred Hospital ICU St George Hospital ICU

St John of God Hospital ICU

St Vincent’s Hospital – Sydney ICU

St Vincent’s Hospital – Melbourne ICU Sunshine Hospital ICU

Tauranga Hospital ICU Tamworth Hospital ICU Tweed Heads Hospital ICU Toowoomba Hospital ICU Townsville Hospital ICU Waikato Hospital ICU Wellington Hospital ICU Wesley Hospital ICU

Western Hospital (Footscray) ICU Wollongong Hospital ICU

Ashford and St Peter’s Hospital ICU

Freeman Hospital (Newcastle upon Tyne) ICU Guy’s and St Thomas’ Hospital ICU

King Abdulaziz Medical City Hospital ICU King Fahad Medical City Hospital ICU King Khalid University Hospital ICU

King’s College Hospital ICU Lewisham Healthcare Trust ICU

Queen Alexandra Hospital (Portsmouth) ICU Queen Elizabeth Hospital (Birmingham) ICU Rigshospitalet ICU

Royal Gwent Hospital ICU

Royal Surrey County Hospital ICU Southampton General Hospital ICU St George’s Healthcare Trust ICU University Hospitals Bristol ICU

# References

1. Walters SJ, Dos Anjos Henriques-Cadby IB, Bortolami O, Flight L, Hind D, Jacques RM, et al. Recruitment and retention of participants in randomised controlled trials: A review of trials funded and published by the United Kingdom Health Technology Assessment Programme. BMJ Open. 2017;7(3).
2. Chlan L, Guttormson J, Tracy MF, Bremer KL. Strategies for overcoming site and recruitment challenges in research studies based in intensive care units. Am J Crit Care. 2009;18(5):410–7.
3. De Backer D, Schortgen F. Physicians declining patient enrollment in clinical trials: What are the implications? Intensive Care Med. 2014;40(1):117–9.
4. Cook D, Arabi Y, Ferguson ND, Heels-Ansdell D, Freitag A, McDonald E, et al.

Physicians declining patient enrollment in a critical care trial: A case study in thromboprophylaxis. Intensive Care Med. 2013;39(12):2115–25.

5. Venkatesh B, Finfer S, Cohen J, Rajbhandari D, Arabi Y, Bellomo R, et al. Adjunctive Glucocorticoid Therapy in Patients with Septic Shock. N Engl J Med. 2018;378(9):797– 808. Available from: <http://www.nejm.org/doi/10.1056/NEJMoa1705835>
6. College of Intensive Care Medicine. Minimum Standards for Intensive Care Units.

College of intensive care medicine. 2011. Available from: <http://www.cicm.org.au/CICM_Media/CICMSite/CICM-> website/Resources/Professional Documents/IC-1-Minimum-Standards-for-Intensive- Care-Units.pdf

7. Ramanan M, Billot L, Rajbhandari D, Myburgh J, Finfer S, Bellomo R, et al. Does

asymmetry in patient recruitment in large critical care trials follow the Pareto principle? Trials. 2020;21(1):1–8.

| Table 1. Site-level characteristics of tertiary and non-tertiary ICUs that participated in the  ADRENAL trial | | | |  |
| --- | --- | --- | --- | --- |
|  | Non-tertiary  (n=35 ICUs) | Tertiary  (n=34 ICUs) | Total  (n=69 ICUs) | P † |
| Volume (admissions per annum), median  (IQR) | 909.5 (633-  1320) | 1737 (1244-  2156) | 1244 (850-  1762) | **<0.001** |
| Director of Research FTE*, median (IQR) | 0 (0-0.05) | 0 (0-0.23) | 0 (0-0.1) | 0.3 |
| Research coordinator FTE, median (IQR) | 1 (1-2) | 2 (1-3) | 2 (1-3) | **0.02** |
| After-hours research coordinator  availability n (%) | 13 (37.1) | 13 (38.2) | 26 (37.7) | 0.9 |
| CTG membership* n (%) | 16 (55.2) | 23 (95.8) | 39 (73.6) | **0.001** |
| Country n (%) |  |  |  | 0.5 |
| Australia | 26 (74.3) | 19 (55.9) | 45 (65.2) |  |
| New Zealand | 3 (8.6) | 5 (14.7) | 8 (11.6) |  |
| Denmark | 0 (0) | 1 (2.9) | 1 (1.5) |  |
| Saudi Arabia | 1 (2.9) | 2 (5.9) | 3 (4.4) |  |
| United Kingdom | 5 (14.3) | 7 (20.6) | 12 (17.4) |  |
| CDR, median (IQR) | 0.09 (0-0.2) | 0.07 (0.02-  0.19) | 0.09 (0.01-  0.19) | 0.8 |

Definitions of abbreviations: ICU= intensive care unit; ADRENAL= Venkatesh et al. ‘*Adjunctive glucocorticoid therapy in patients with septic shock’* Trial; IQR= interquartile range; FTE= full-time equivalent; CTG= Australia New Zealand Intensive Care Society Clinical Trials Group; CDR= clinician decline rate

- Director of Research FTE and CTG membership data is for Australia and New Zealand sites only

^†^ p values presented in this column have been generated from Fisher’s exact test for categorical variables and Mann-Whitney U tests for continuous variables. Bold p-values are statistically significant

| Table 2. Multivariable logistic regression for eligible patients being declined for  randomization by clinicians in the ADRENAL trial | | |
| --- | --- | --- |
| **All sites model (n=5329)** | **Odds Ratio (95%**  **CI)** | **P*** |
| Tertiary ICU (versus non-tertiary) | 0.89(0.35-2.31) | 0.8 |
| ANZ site (yes versus no) | 1.86(0.62-5.59) | 0.3 |
| Volume (per 1000 admissions/year) | 1.14(0.66-1.98) | 0.6 |
| First author site (yes versus no) | 0.29(0.03-3.13) | 0.3 |
| Management committee site (yes versus no) | 1.27(0.41-3.97) | 0.7 |
| Research coordinator FTE (per 1 FTE) | 0.99(0.73-1.34) | 1 |
| After-hours research coordinator availability (yes versus  no) | 0.70(0.31-1.56) | 0.4 |
| **ANZ sites only model (n=4291)** | **Odds Ratio (95%**  **CI)** | **p** |
| Tertiary ICU (versus non-tertiary) | 0.52(0.17-1.59) | 0.3 |
| Volume (per 1000 admissions/year) | 1.15(0.52-2.55) | 0.7 |
| CTG membership (yes versus no) | 2.86(0.92-8.88) | 0.1 |
| First author site (yes versus no) | 0.27(0.03-2.61) | 0.3 |
| Management committee site (yes versus no) | 1.87(0.56-6.19) | 0.3 |
| Research coordinator FTE (per 1 FTE) | 0.99(0.63-1.55) | 1 |
| After-hours research coordinator availability (yes versus  no) | 0.76(0.34-1.71) | 0.5 |
| Director of Research FTE (per 1 FTE) | 0.84(0.24-2.97) | 0.8 |

Definitions of abbreviations: ICU= intensive care unit; ADRENAL= Venkatesh et al. ‘*Adjunctive glucocorticoid therapy in patients with septic shock’* Trial; ANZ= Australia and New Zealand; FTE= full- time equivalent; CTG= Australia New Zealand Intensive Care Society Clinical Trials Group

- Each effect estimate listed in this table has been generated from one of two multivariable mixed effects model (“All sites model” and “ANZ sites only model”) i.e., they are adjusted for all other variables listed in the table.
