# Supplementary material for: An evaluation of factors that may influence clinicians’ decisions not to enroll eligible patients into randomized trials in critical care
Source: PLoS One. 2021 Jul 27;16(7):e0255361. doi: 10.1371/journal.pone.0255361 (PMC8315530; doi:10.1371/journal.pone.0255361)
Supplement: Attachment — Submitted filename: Responses to reviewers.docx [file pone.0255361.s003.docx]

Dear Prof Linda Maerz,

Thank you for your thoughtful review and for giving us an opportunity to submit a revised manuscript. We have addressed all the points raised by the Reviewers and Editors. Our responses are highlighted in yellow after each query. Tracked and clean versions of the manuscript are attached to the submission as requested.

We look forward to hearing the outcome of your review.

Yours sincerely,

Mahesh Ramanan on behalf of all authors

Date: 11 May 2021

**Reviewer 1**

*It is logical to characterize the study sites as having trainees and research support to attempt to identify reasons why clinicians may not include eligible patients in a clinical trial.
However, the study is a bit superficial:
First - Only one trial (ADRENAL), though a large trial, was included in the analysis. Any trial specific factors cannot be isolated or assessed. In other words, it is difficult to say if the included research sites had specific protocol reasons to decline eligible patients. Including other trials would potentially provide additional clarity.*

Thank you for your review and comments. It is true that trial-specific factors cannot be assessed with our study. Having access to a larger group of trials with similar data would be ideal. However, this was not the case for our study as we did not have access to such data. We have included a statement in the discussion to this effect.

*Second - Additional site-level characteristics are needed. It is a big jump to say that because there is no correlation between a site being academic or having research support that the clinicians are likely concerned about a lack of equipoise.
While it helps that the ADRENAL trial allowed deferred consent, the authors' conclusion is still based on speculation.
For example, in how many other trials of this size did the sites participate?
Were the rates of declined eligible patients similar for all studies?*

That particular statement with regards to deferred consent and clinician equipoise is indeed speculative, as we have acknowledged in the text. We have added some further discussion to clarify this point.

The data concerning other trials (‘competing trials’) and particularly the rates of CDR in these trials was not available to us. The ADRENAL trial protocol did allow for co-enrollment with several other trials, so it is possible that there were sites which were recruiting in competing trials.

**Reviewer 2**

*The methods are described with adequate detail. The study is probably underpowered as indicated by the wide confidence intervals reported for many of the variables. Realizing that that the sample size was fixed by what was available in the RCT data base I did not see a description of how adequate power was estimated. Having an estimate of the sample size required to detect differences in outcome could inform future studies and place the current results in perspective.*

Thank you for your review and for raising the important issue of statistical power. We did not have a baseline clinician decline rate which could be used for power calculations, hence, a priori calculations were not performed. Our results may be used to inform sample size calculations for future studies.

We have elected not to perform post hoc calculations of power as there is significant controversy in the biostatistics literature regarding the validity of such calculations. A recent publication by Zhang et al (Zhang Y, Hedo R, Rivera A, et al. Post hoc power analysis: is it an informative and meaningful analysis? General Psychiatry 2019;32:e100069. doi: 10.1136/gpsych-2019-100069) had the following concluding statements:

*“Power analysis is an indispensable component of planning clinical research studies. However, when used to indicate power for outcomes already observed, it is not only conceptually flawed but also analytically misleading. Our simulation results show that such power analyses do not indicate true power for detecting statistical significance, since post hoc power estimates are generally variable in the range of practical interest and can be very different from the true power.*

*In this report, we focus on the relatively simple statistical model for comparing two population means of continuous outcomes. The same considerations and conclusions also apply to non-continuous outcomes and more complex models such as regression. In general, post hoc power analyses do not provide sensible results.”*

**Responses to Comments**

Page 3

*What does this mean?*

This should be tertiary status which is defined in the Methods section.

Page 4

*Requires a space between “We” and “defined”*

This has been inserted.

*What is the rationale for derivation of these ICU-level variables? Wouldn’t ICU staffing also be relevant? For instance—full-versus part-time intensivist staffing; physician versus advanced practice provider staffing; trainee presence; number of patients per intensivist, etc.*

We selected these variables based on availability and relevance to Australia and New Zealand Intensive Care practice. All ICUs included in the trial in Australia and New Zealand are staffed by specialist Intensivists on-site during the day and on-call at night with on-site trainee presence all 24 hours. There are no advanced practice providers or nurse practitioners in ANZ ICUs. The seniority of trainee presence (i.e. basic trainee, advanced trainee, fellow) is determined by the College of Intensive Care Medicine Level as described earlier in the Methods section. The number pf patients looked after by intensivists in ANZ follow the recommended CICM guidelines as these sites are accredited by the CICM for the purpose of training. We chose volume of admissions as a marker of ICU size, and the other variables selected were resourcing variables that have a direct link to research i.e. availability of research coordinators, director of research etc.

Page 5

*Percentage?*

Yes. This has been changed.

Page 6

*Please provide more detail pertaining to the rationale of this speculation.*

Yes. We have now provided this detail in the preceding paragraph.

*Need a space between “of” and equipoise.*

This sentence has been removed.

Page 7

*Need space between “but” and “have”*

This has been inserted.

*If the study is underpowered, please explain why the conclusions are valid.*

Thanks. We have added further to the paragraph to explain that the findings are exploratory but also why they hold validity.

*Please define this.*

We have shortened the sentence for clarity.

Page 8

*Need a space between “on” and “enrollment”*

This has been inserted.
